# Supplementary material for: A field-deployable diagnostic assay for the visual detection of misfolded prions
Source: Sci Rep. 2022 Jul 18;12:12246. doi: 10.1038/s41598-022-16323-y (PMC9293997; doi:10.1038/s41598-022-16323-y)
Supplement: Supplementary file 1 — Supplementary Information. [file 41598_2022_16323_MOESM1_ESM.docx]

Supporting Information

**A Field-Deployable Diagnostic Assay**

**for the Visual Detection of Misfolded Prions**

Peter R. Christenson^1,2^, Manci Li^2,3^, Gage Rowden^2,3^, Marc D. Schwabenlander^2,3^,

Tiffany M. Wolf^2,4^, Sang-Hyun Oh^1,2^, Peter A. Larsen^2,3*^

# ^1^Department of Electrical and Computer Engineering, University of Minnesota, Minneapolis, MN 55455, United States

^2^Minnesota Center for Prion Research and Outreach, University of Minnesota, St. Paul, MN 55108, United States

^3^Department of Veterinary and Biomedical Sciences, University of Minnesota, St. Paul, MN 55108, United States

^4^Department of Veterinary and Population Medicine, University of Minnesota, St. Paul, MN 55108, United States

*E-mail: [plarsen@umn.edu](mailto:plarsen@umn.edu) and [sang@umn.edu](mailto:sang@umn.edu)

**Supplemental Results**

**
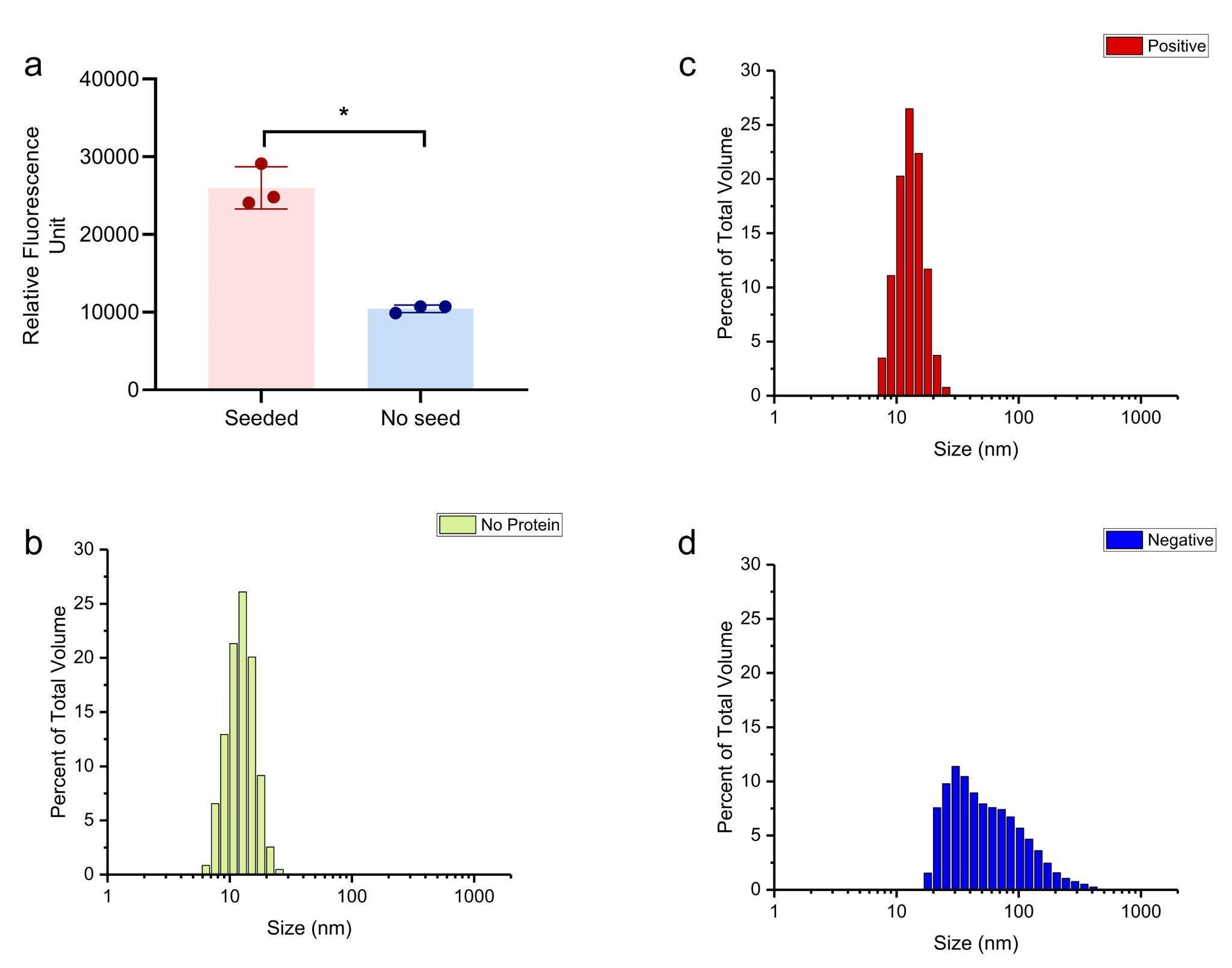
**

**Supplementary Figure 1.** ThT fluorescence, light absorbance, and average particle size measurements for native and misfolded recombinant hamster prion protein. **a.** The relative fluorescence units (ThT fluorescence) of post QuIC solutions containing misfolded protein seeds and solutions without misfolded protein seeds. **b.** Distribution of DLS readings of AuNP solution with no protein added. Reported as percent of total volume of AuNPs present. **c.** Distribution of DLS readings of AuNP solution with misfolded (positive) rPrP. **d.** Distribution of DLS readings of AuNP solution with native (negative) rHaPrP. *, p-value < 0.05, error bars show standard deviation.

| **Supplementary Table 1. Metadata of Examined Wild White-Tailed Deer RPLN.** MNPRO = Minnesota Center for Prion Research and Outreach. RPLN = medial retropharyngeal lymph node. Official CWD test result indicates the RPLN was independently examined using ELISA and/or IHC testing at Colorado State University (see Schwabenlander et al. 2021, citation 27 in main text). ND=Not Detected. | | | | | |
| --- | --- | --- | --- | --- | --- |
| **Animal MNPRO ID** | **Age** | **Sex** | **Official CWD Test Result** | **RT-QuIC**  **Result** | **MN-QuIC Results** |
| 835 | Adult | Female | Positive | Positive | Positive |
| 826 | Adult | Male | Positive | Positive | Positive |
| 830 | Adult | Female | Positive | Positive | Positive |
| 836 | Adult | Male | Positive | Positive | Positive |
| 922 | Adult | Female | ND | ND | ND |
| 924 | Adult | Female | ND | ND | ND |
| 925 | Fawn | Female | ND | ND | ND |
| 926 | Fawn | Female | ND | ND | ND |

**Supplementary Table 2. Metadata of Examined Wild White-Tailed Deer in Field Test.** RPLN=medial retropharyngeal lymph node. Official CWD test result indicates the RPLN was independently examined using ELISA and/or IHC testing at Colorado State University (see Schwabenlander et al. 2021, citation 27 in main text). ND=Not Detected

| **Animal ID** | **Age** | **Sex** | **Official CWD Test Results for RPLN** | **MN-QuIC Results** |
| --- | --- | --- | --- | --- |
| 197190 | Female | Yearling | Positive | Positive |
| 197270 | Female | Fawn | ND | ND |
| 197273 | Female | Adult | ND | ND |
| 197264 | Female | Adult | ND | ND |
| 197266 | Female | Adult | ND | ND |
| 197265 | Female | Adult | ND | ND |
| 197267 | Male | Fawn | ND | ND |
| 197268 | Female | Fawn | ND | ND |
| 197269 | Female | Adult | ND | ND |
| 197271 | Female | Adult | ND | ND |
| 197272 | Male | Fawn | ND | ND |
| 197225 | Male | Yearling | Positive | Positive |
| 197065 | Male | Adult | Positive | Positive |

**Supplementary Table 3. Metadata of examined wild white-tailed deer (RPLN; blinded analyses).** MNPRO = Minnesota Center for Prion Research and Outreach. RPLN=medial retropharyngeal lymph node. Official CWD test result indicates the RPLN was independently examined using ELISA and/or IHC testing at Colorado State University (see Schwabenlander et al. 2021, citation 27 in main text). ND=Not Detected. For MN-QuIC there were 8 replicates for each tissue.

| **Sample Process ID** | **Animal MNPRO ID** | **Age** | **Sex** | **Official CWD Test Results for RPLN** | **MN-QuIC Results** | **Number of Red replicates** |
| --- | --- | --- | --- | --- | --- | --- |
| 1384 | 929 | Adult | M | Not Detected | Not Detected | 0 |
| 1380 | 925 | Fawn | F | Not Detected | Not Detected | 0 |
| 1386 | 931 | Fawn | F | Not Detected | Not Detected | 0 |
| 1381 | 926 | Fawn | F | Not Detected | Not Detected | 0 |
| 1389 | 934 | Adult | F | Not Detected | Not Detected | 0 |
| 1385 | 930 | Fawn | F | Not Detected | Not Detected | 0 |
| 1383 | 928 | Yearling | F | Not Detected | Not Detected | 0 |
| 1378 | 922 | Adult | F | Not Detected | Not Detected | 0 |
| 1390 | 935 | Yearling | M | Not Detected | Not Detected | 0 |
| 1392 | 937 | Yearling | F | Not Detected | Not Detected | 0 |
| 954 | 1137 | Fawn | F | Not Detected | Not Detected | 0 |
| 1391 | 936 | Fawn | M | Not Detected | Not Detected | 0 |
| 1395 | 940 | Yearling | F | Not Detected | Not Detected | 0 |
| 1388 | 933 | Fawn | M | Not Detected | Not Detected | 0 |
| 1394 | 939 | Yearling | M | Not Detected | Not Detected | 0 |
| 1393 | 938 | Yearling | F | Not Detected | Not Detected | 0 |
| 1387 | 932 | Fawn | F | Not Detected | Not Detected | 0 |
| 958 | 1138 | Adult | F | Not Detected | Not Detected | 1 |
| 1382 | 927 | Adult | F | Not Detected | Not Detected | 0 |
| 1379 | 924 | Adult | F | Not Detected | Not Detected | 1 |
| 923 | 829 | Adult | M | Positive | Not Detected | 2 |
| 929 | 835 | Adult | F | Positive | Positive | 8 |
| 925 | 831 | Adult | F | Positive | Positive | 4 |
| 927 | 833 | Adult | F | Positive | Positive | 8 |
| 914 | 310 | adult | M | Positive | Positive | 8 |
| 926 | 832 | Yearling | M | Positive | Not Detected | 2 |
| 922 | 828 | Adult | F | Positive | Positive | 8 |
| 928 | 834 | Adult | F | Positive | Positive | 7 |
| 921 | 827 | Adult | M | Positive | Positive | 8 |
| 1376 | 318 | ADULT | M | Positive | Positive | 4 |
| 1374 | 316 | Yearling | M | Positive | Positive | 8 |
| 1377 | 319 | Yearling | M | Positive | Positive | 8 |
| 1372 | 302 | Adult | M | Positive | Positive | 8 |
| 1375 | 317 | Adult | M | Positive | Positive | 8 |
| 930 | 836 | Adult | M | Positive | Positive | 8 |
| 931 | 837 | Adult | F | Positive | Positive | 8 |
| 1373 | 315 | Adult | F | Positive | Positive | 8 |
| 932 | 838 | Adult | F | Positive | Positive | 8 |
| 920 | 826 | Adult | M | Positive | Positive | 8 |
| 924 | 830 | Adult | F | Positive | Positive | 5 |

| **Supplementary Table 4. Metadata of examined wild white-tailed deer tonsils.** MNPRO = Minnesota Center for Prion Research and Outreach. RPLN=medial retropharyngeal lymph node. Official CWD test result indicates the RPLN was independently examined using ELISA and/or IHC testing at Colorado State University (see Schwabenlander et al. 2021, citation 27 in main text). ND=Not Detected | | | | | | |
| --- | --- | --- | --- | --- | --- | --- |
| **Sample Process ID** | **Animal MNPRO ID** | **Age** | **Sex** | **Official CWD Test Results for RPLN** | **RT-QuIC**  **Results for Tonsil** | **MN-QuIC Results** |
| 000734 | 835 | Adult | Female | Positive | Positive | Positive |
| 000735 | 832 | Yearling | Male | Positive | Positive | Positive |
| 000736 | 826 | Adult | Male | Positive | Positive | Positive |
| 000737 | 838 | Adult | Female | Positive | Positive | Positive |
| 000738 | 830 | Adult | Female | Positive | Positive | Positive |
| 000739 | 831 | Adult | Female | Positive | Positive | Positive |
| 000740 | 833 | Adult | Female | Positive | Positive | Positive |
| 000741 | 834 | Adult | Female | Positive | Positive | Positive |
| 000742 | 498 | Yearling | Male | ND | ND | ND |
| 000743 | 836 | Adult | Male | Positive | Positive | Positive |
| 000744 | 828 | Adult | Female | Positive | Positive | Positive |
| 000745 | 065 | Adult | Female | ND | ND | ND |
| 000746 | 067 | Adult | Female | ND | ND | ND |
| 000747 | 091 | Adult | Female | ND | ND | ND |
| 000748 | 093 | Adult | Female | ND | ND | ND |
| 000749 | 230 | Fawn | Female | ND | ND | ND |
| 000750 | 232 | Adult | Female | ND | ND | ND |
| 000751 | 283 | Fawn | Male | ND | ND | ND |
| 000752 | 415 | Fawn | Female | ND | ND | ND |
| 000753 | 431 | Adult | Male | ND | ND | ND |


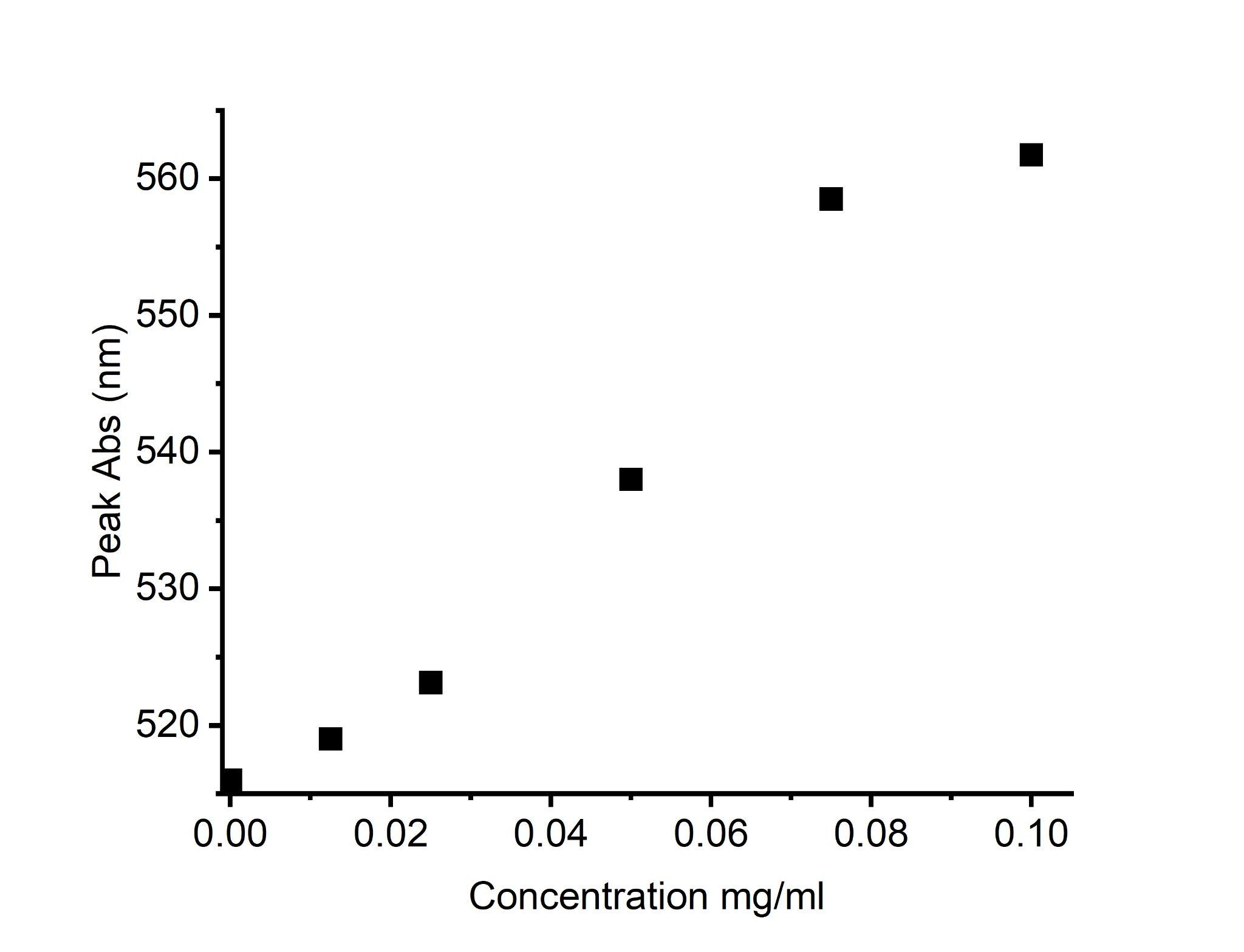


**Supplementary Figure 2.** Wavelength of peak absorbance of AuNPs spiked with various concentrations of native rPrP substrate in standard mastermix buffer.

**Supplementary Table 5. Summary of data sets examined herein:** RPLN=medial retropharyngeal lymph node. Official CWD test result indicates the RPLN was independently examined using ELISA and/or IHC testing at Colorado State University (see Schwabenlander et al. 2021, citation 27 in main text). ND=Not Detected. CI= Wilson score 95% confidence interval.

| **Data Set** | **Tissue type** | **Number of Positive Animals/ tissues via ELISA/ IHC** | **Number of Not Detected Animals/ tissues via ELISA/ IHC** | **MN-QuIC Sensitivity compared to ELISA/ IHC** | **MN-QuIC Specificity compared to ELISA/ IHC** | **SensitivityLower- Upper CI** | **Specificity Lower- Upper CI** |
| --- | --- | --- | --- | --- | --- | --- | --- |
| Table S1 | RPLN | 4 | 4 | 100% | 100% | 0.5101- 1.000 | 0.5101- 1.000 |
| Table S2 (Field Test) | Various lymph tissue | 3 | 10 | 100% | 100% | 0.4385- 1.000 | 0.7225- 1.000 |
| Table S3 | RPLN | 20 | 20 | 90% | 100% | 0.6990- 0.9721 | 0.8389- 1.000 |
| Table S4 | Palatine Tonsils | 10 | 10 | 100% | 100% | 0.7225- 1.000 | 0.7225- 1.000 |


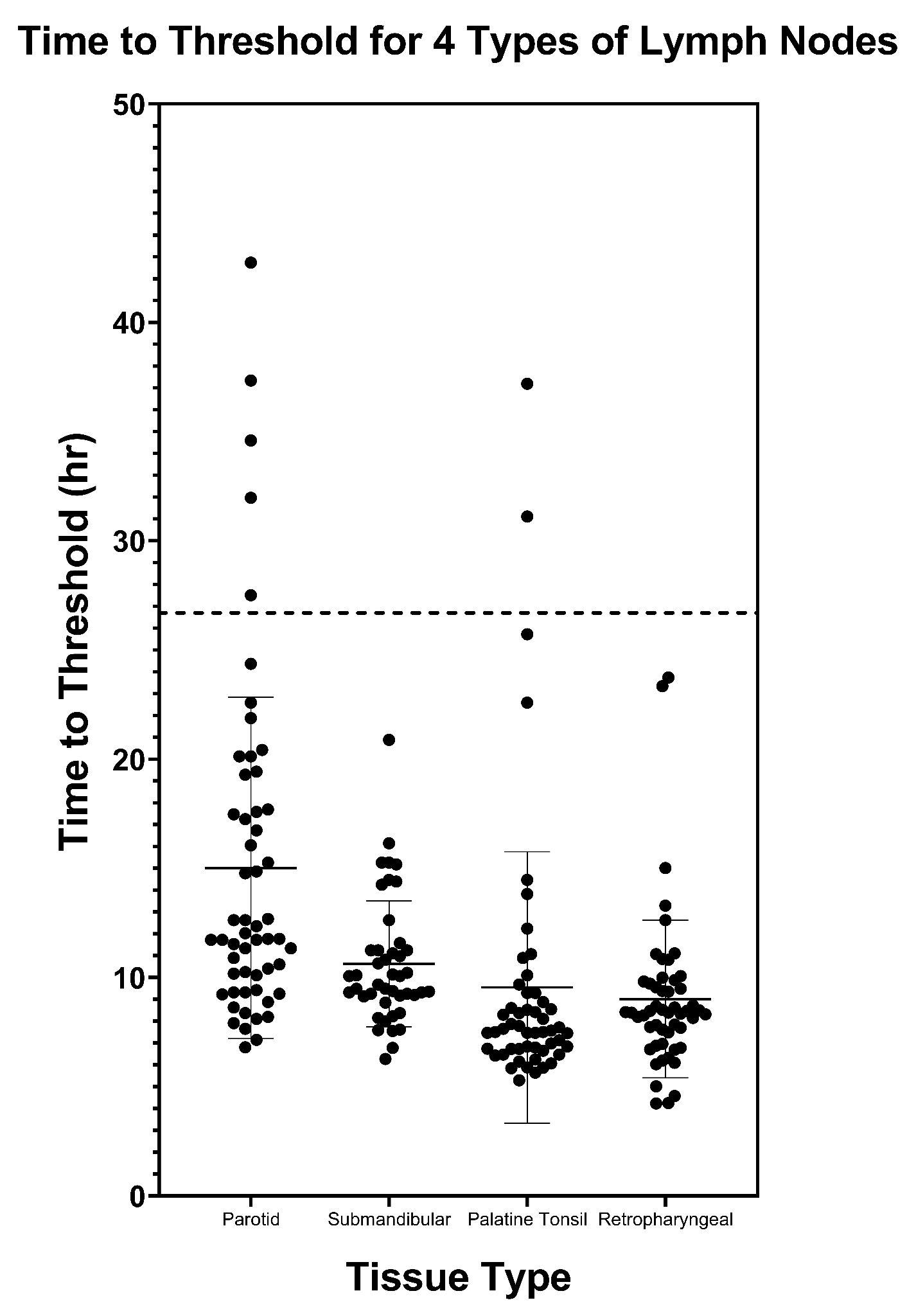


**Supplementary Figure 3:** Prion seeding activity as shown by time (in hours) to Thioflavin-T fluorescence threshold via RT-QuIC reactions for various CWD positive white-tailed deer lymph tissues. Dashed line identifies a 24hr threshold used for MN-QuIC analyses of retropharyngeal lymph nodes.
